# ErbB-3 activation by NRG-1β sustains growth and promotes vemurafenib resistance in BRAF-V600E colon cancer stem cells (CSCs)

**DOI:** 10.18632/oncotarget.4642

**Published:** 2015-06-25

**Authors:** Pramudita R. Prasetyanti, Emily Capone, Daniela Barcaroli, Daniela D'Agostino, Silvia Volpe, Antonina Benfante, Sander van Hooff, Valentina Iacobelli, Cosmo Rossi, Stefano Iacobelli, Jan Paul Medema, Vincenzo De Laurenzi, Gianluca Sala

**Affiliations:** ^1^ Laboratory for Experimental Oncology and Radiobiology (LEXOR), Center for Experimental and Molecular Medicine, Academic Medical Center (AMC), Amsterdam, The Netherlands; ^2^ Cancer Genomics Center, The Netherlands; ^3^ Dipartimento di Scienze Mediche, Orali e Biotecnologiche, University “G. d'Annunzio” Chieti-Pescara, Centro Studi sull'Invecchiamento, Ce.S.I., Chieti, Italy; ^4^ Department of Surgical and Oncological Sciences, Cellular and Molecular Pathophysiology Laboratory, University of Palermo, Palermo, Italy; ^5^ Department of Gynecology and Obstetrics, La Sapienza University of Rome, Rome, Italy; ^6^ MediaPharma s.r.l., Chieti, Italy

**Keywords:** ErbB-3, vemurafenib, NRG-1β, colon cancer stem cells

## Abstract

Approximately 5-10% of metastatic colorectal cancers harbor a BRAF-V600E mutation, which is correlated with resistance to EGFR-targeted therapies and worse clinical outcome. Vice versa, targeted inhibition of BRAF-V600E with the selective inhibitor PLX 4032 (Vemurafenib) is severely limited due to feedback re-activation of EGFR in these tumors. Mounting evidence indicates that upregulation of the ErbB-3 signaling axis may occur in response to several targeted therapeutics, including Vemurafenib, and NRG-1β-dependent re-activation of the PI3K/AKT survival pathway has been associated with therapy resistance.

Here we show that colon CSCs express, next to EGFR and ErbB-2, also significant amounts of ErbB-3 on their membrane. This expression is functional as NRG-1β strongly induces AKT/PKB and ERK phosphorylation, cell proliferation, clonogenic growth and promotes resistance to Vemurafenib in BRAF-V600E mutant colon CSCs. This resistance was completely dependent on ErbB-3 expression, as evidenced by knockdown of ErbB-3. More importantly, resistance could be alleviated with therapeutic antibody blocking ErbB-3 activation, which impaired NRG-1β-driven AKT/PKB and ERK activation, clonogenic growth *in vitro* and tumor growth in xenograft models. In conclusion, our findings suggest that targeting ErbB-3 receptors could represent an effective therapeutic approach in BRAF-V600E mutant colon cancer.

## INTRODUCTION

Colorectal cancer (CRC) accounts for almost 10% of all cancers and it is the third most common cancer worldwide and still a major cause of cancer-related deaths [1]. The human ErbB receptor family, including EGFR (ErbB-1/HER-1), ErbB-2 (HER-2), ErbB-3 (HER-3) and ErbB-4 (HER-4) have been documented to play a fundamental role in the development and progression of several malignancies [2]. As a consequence, a multitude of targeted therapeutics have been developed to block the activity of these receptors [3] and over the past decades the development of EGFR targeted therapeutics has improved the clinical outcome of metastatic CRC patients [4]. Despite these encouraging results, the reason why some patients respond to treatment, while others don't remains poorly understood. Understanding the mechanism(s) of resistance to EGFR targeted agents is therefore crucial to gain a significant improvement in survival of CRC patients.

Among the molecular pathways involved in CRC progression and therapy resistance, the RAS-RAF-MEK-ERK axis plays a crucial role. Indeed, lack of response to agents targeting EGFR in *KRAS* wild-type patients can result from *BRAF* mutations at codon 600, which occur in 8-10% of metastatic CRC. Metastatic patients harboring *BRAF* mutations display an extremely poor prognosis, with a median survival of about 10 months [5, 6]. Therapies targeting mutated BRAF have been developed and are currently used in specific malignancies. As an example, Vemurafenib (PLX 4032), a small molecule inhibiting specifically mutant BRAF-V600E, has been successfully used in metastatic melanoma patients [7, 8]. However, no significant benefit from Vemurafenib use has been observed in CRC patients [9]. In addition, accumulating evidence suggests that, next to pathway mutations, other receptor/ligand pairs may substitute the loss of EGF/EGFR signaling and play a crucial role in anti-EGFR therapy resistance. As an example, HGF/c-Met activation has been suggested to result in resistance to anti-EGFR based therapies [10]. In addition, it has been shown that high expression of ErbB-3 correlates to worse outcome in CRC [11, 12]. Moreover, NRG-1β, the ligand for ErbB-3, is released by tumor-associated stromal cells and has been suggested to promote CRC progression as well as compensate for loss of EGF/EGFR signaling [13].

Here we analyzed the role of ErbB-3/NRG-1β signaling on primary cultures derived from patients with either mutant or wild-type BRAF-V600E CRC. These primary cultures contain both cancer stem cells (CSCs) and more differentiated cells and we observed that NRG-1β sustains proliferation and cancer stemness in both wild-type and BRAF-V600E mutant CSCs by activating the PI3K/AKT and ERK signaling axes. We also demonstrate that NRG-1β, in addition to EGF, can effectively induce escape from Vemurafenib therapy in BRAF-V600E mutant colon CSC cultures. Finally, we show that targeting ErbB-3 receptors *in vivo*, with a specific monoclonal antibody, significantly delays tumor growth of BRAF-V600E mutant colon cancer xenografts. Together, our data underline the importance of NRG-1β in CRC stimulation and resistance to BRAF-V600E targeted therapy.

## RESULTS

### Vemurafenib cytotoxic activity is blocked by EGF

**Figure 1:**
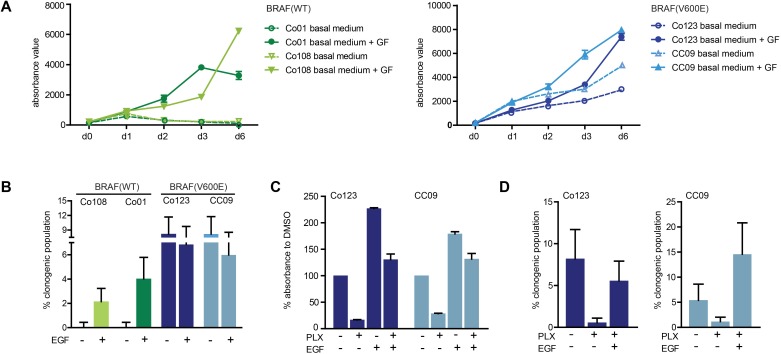
Effect of growth factors on wild-type and V600E-BRAF mutated colon CSCs **A.** Cell proliferation was determined in wild-type (left panel) and BRAF mutant cells (right panel) in the presence or absence of growth factors (EGF and bFGF) by Cell Titer-Blue assay at the indicated time points. **B.** Basal or EGF-induced clonogenicity was assessed by limiting dilution assay in all cell lines. Proliferation evaluated by Cell Titer-Blue assay after 5 days of culture **C.** and clonogenicity evaluated by limiting dilution assay **D.** in BRAF mutant (Co123 and CC09) cells treated with Vemurafenib (1 μM) in the presence or absence of EGF. Data shown represent mean +/− SD from triplicate samples.

We first evaluated the effect of growth factors, EGF and bFGF, on proliferation and clonogenicity of a panel of wild-type and BRAF-V600E mutated patient-derived primary cultures of CRC. As shown in Figure 1A and 1B, cells carrying the BRAF-V600E mutation displayed a significantly lower dependence on growth factors as compared to wild-type cells, both in terms of proliferation rate and clonogenicity, confirming that this mutation was sufficient to sustain growth of these cells. Next we investigated the effect of Vemurafenib on two BRAF-V600E mutated cell lines. Proliferation (Figure 1C) and clonogenicity (Figure 1D) of cells cultured in the absence of growth factors was strongly inhibited by the drug; however this block was completely rescued by addition of exogenous EGF, thus confirming the previously reported role of EGFR signaling [14] in resistance to Vemurafenib.

This strong inhibition of growth by Vemurafenib was confirmed using gene expression arrays, which revealed that a total of ~400 genes were significantly differentially expressed between controls and Vemurafenib treated samples (Supplementary Figure 1A). Analysis of the biological pathways affected indicated a strong decrease in cell cycle/mitosis related genes in treated cells (Supplementary Figure 1B). Since previous observations suggested that EGFR activation counteracts Vemurafenib-induced growth arrest [14] we analyzed whether BRAF inhibition had an effect on the expression of EGFR and its close relatives (ErbB family). While no significant changes in EGFR expression were observed between control and Vemurafenib treated samples, we detected a significant increase in the expression of ErbB-3 (Supplementary Figure 1C). This was confirmed by western blotting for the different receptors (Supplementary Figure 1D). Combined this suggested that up-regulation of the expression of this gene may constitute an escape mechanism from Vemurafenib.

### NRG-1β sustains proliferation and counteracts Vemurafenib activity in BRAF-V600E CRC CSCs

**Figure 2:**
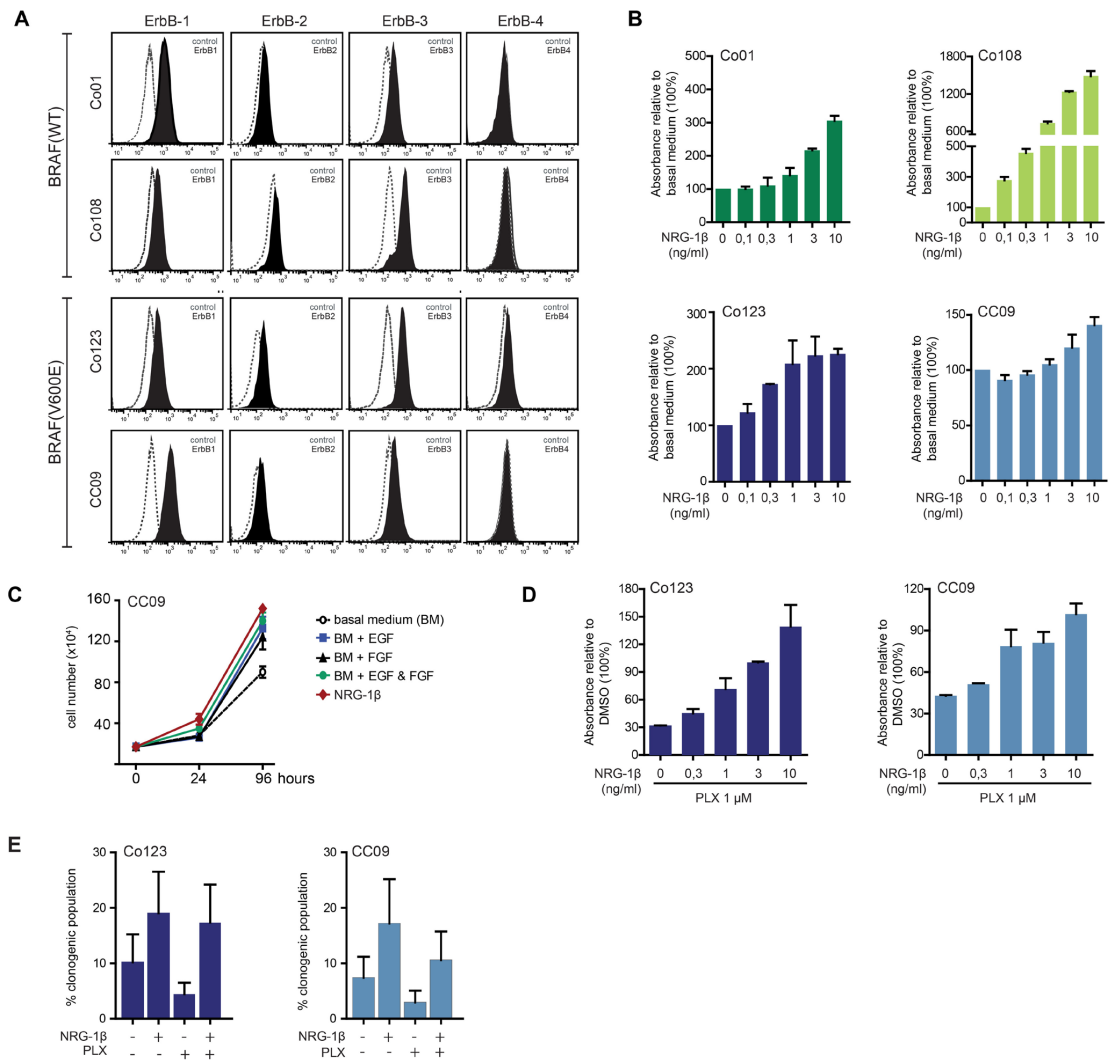
NRG-1β sustains proliferation of colon CSCs and reverts Vemurafenib antitumor effects **A.** Surface expression level of ErbB receptors analyzed by FACS in both wt and BRAF mutant cells. **B.** Cell proliferation analysis of colon CSCs stimulated with increasing doses of NRG-1β evaluated by Cell Titer-Blue assay after 5 days of culture. **C.** CC09 cells were cultured in presence or absence of growth factors (EGF: 20 ng/ml; bFGF: 10 ng/ml; NRG-1β: 10 ng/ml) as indicated and proliferation assessed by cell count. **D.** Cell proliferation analysis evaluated by Cell Titer-Blue assay in BRAF mutant cells treated for 5 days with Vemurafenib in the presence of increasing amount of NRG-1β. **E.** Clonogenicity was evaluated by limiting dilution assay on BRAF mutant cells treated with Vemurafenib (1 μM) in presence or absence of NRG-1β.

NRG-1β, the predominant ErbB-3 ligand, has been shown to be released in the tumor microenvironment by stromal cells and to promote CRC progression through ErbB-3 mediated PI3K/AKT activation [13]. To determine whether ErbB-3 could play a role in colon CSC stimulation, we first analyzed ErbB receptors surface expression in our panel of patient-derived colon CSCs. Both wild-type and BRAF-V600E mutated cells expressed EGFR, ErbB-2 and ErbB-3, but not ErbB-4 (Figure 2A).

We then investigated whether NRG-1β was able to promote proliferation, clonogenicity and PI3K/AKT activation in these cells. Interestingly, stimulation of wild-type and BRAF-V600E mutated cells with the ligand, dose-dependently promoted cell proliferation. Similarly to what was observed with other growth factors (EGF and bFGF), this effect was more pronounced in wild-type cells compared to those carrying the BRAF-V600E mutation (Figure 2B). Of note, NRG-1β appeared to be as potent as EGF in promoting cell proliferation when used alone (Figure 2C).

To determine whether Vemurafenib-induced ErbB-3 increase resulted in a potential escape mechanism, we investigated whether activation of ErbB-3 by exogenous NRG-1β could counteract the anti-proliferative effect of Vemurafenib in BRAF-V600E mutated cells. To this end, Co123 and CC09 cells were exposed to Vemurafenib in the absence or presence of increasing concentrations of NRG-1β. As shown in Figure 2D, we found that NRG-1β dose-dependently reduced the inhibitory activity of the BRAF inhibitor. Similar results were obtained in clonogenic assays, indicating that NRG-1β ligand provides an effective escape mechanism from blockage of BRAF-V600E (Figure 2E).

### ErbB-3 is required for Vemurafenib rescue induced by exogenous NRG-1β

**Figure 3:**
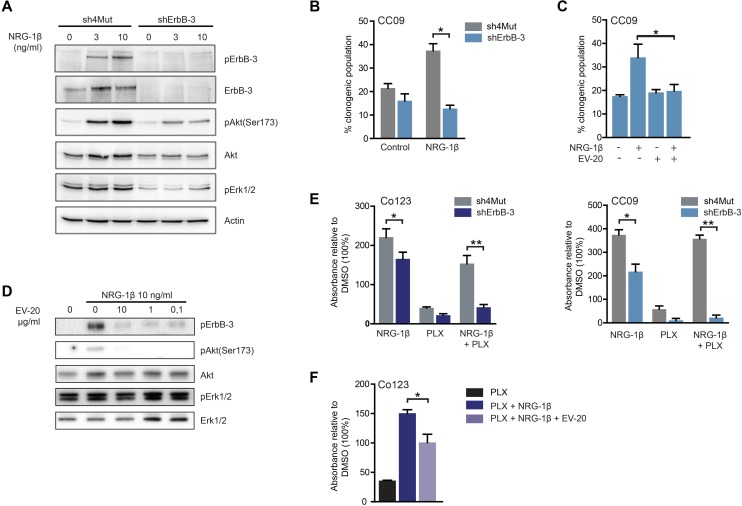
ErbB-3 is required for NRG-1**β**-dependent escape to Vemurafenib **A.** Immunoblot analysis of total and phosphorylated of ErbB-3 receptor and downstream signaling pathways evaluated in CC09 cells silenced for ErbB-3 (shErbB-3) as compared to same cells stably infected with the control vector (sh4Mut). After 24 hrs of growth factors deprivation, cells were stimulated with 10 ng/ml of NRG-1β for 5 minutes, then cell lysates were blotted as indicated. NRG-1β (10 ng/ml) stimulated clonogenicity was determined by limiting dilution assay in ErbB-3 silenced CC09 cells **B.** or in CC09 cells treated with 10 μg/ml of the anti-ErbB-3 antibody EV20 **C. D.** Co123 cells were cultured overnight in absence of growth factors and then treated with increasing doses of EV20 for 8 hrs before stimulation with NRG-1β (10 ng/ml) for 5 minutes; cell lysates were blotted as indicated. **E.** The rescue effect of NRG-1β (10 ng/ml) from Vemurafenib treatment (1 μM) was evaluated in CC09 and Co123 cells silenced for ErbB-3 (shErbB-3) as compared to control cells (sh4Mut). Proliferation was assessed by Cell Titer-Blue assay after 6 days of treatment either with NRG-1β (10 ng/ml) or Vemurafenib (1 μM) or a combination of the two. **F.** Proliferation, evaluated as in E, of BRAF mutant Co123 cells treated with Vemurafenib (1 μM) in the presence or absence of NRG-1β and EV20 (10 μg/ml). Results are expressed as mean +/− SD of two **B.** or three (C, E and F) independent experiments.* = *p* < 0.05, ** = *p* <0.01 (*t*-test).

We next investigated the role of ErbB-3 receptor in NRG-1β-dependent resistance to Vemurafenib. To this end we stably silenced ErbB-3 expression in the two BRAF-V600E mutated cell lines. Loss of ErbB-3 expression caused a marked impairment of ErbB-3, AKT and ERK phosphorylation upon ligand stimulation (Figure 3A and data not shown). Further, NRG-1β-dependent sphere forming ability was impaired in receptor silenced cells as compared to control (Figure 3B). Similarly, cells treated with the ErbB-3 blocking antibody, EV20, showed a marked reduction of ligand-induced clonogenicity and activation of AKT and ERK (Figure 3C and 3D). Moreover, we found that ErbB-3 knock-down or its blockage by antibody treatment (EV20) abrogated the NRG-1β dependent rescue from Vemurafenib (Figure 3E and 3F). Consistently, NRG-1β dependent ErbB-3 activation is increased in Vemurafenib treated cells and can be blocked by EV20 (Supplementary Figure 2). Combined, these observations indicate that the NRG-1β/ErbB-3 axis is an effective mechanism of escape from Vemurafenib treatment, in addition to the already reported activation of EGFR.

**Figure 4:**
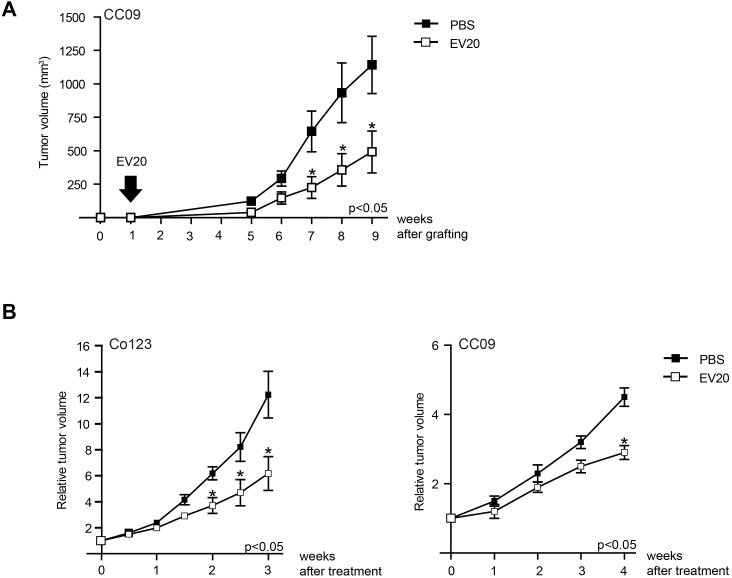
Treatment with anti-ErbB-3 antibody results in delay of V600E-BRAF tumor growth Tumor growth was assessed as described in Materials and Methods. **A.** Mice injected with CC09 (2.5 × 10^5^) cells were divided in two groups one week after the engraftment. The treated group received 10 mg/kg twice weekly of EV20 in PBS whereas the control group received PBS only. Arrow indicates the start of treatment. **B.** Mice injected with either CC09 (2.5 × 10^5^) or Co123 (1×10^6^) cells and divided into size homogeneous groups once established tumors had reached the approximate Volume of 100 mm^3^, then treated with 10 mg/kg twice weekly of EV20. Control groups were treated with PBS. Results are expressed as Relative Tumor Volume, *p* values were determined by Student's *t* test and considered significant for *p* < 0.05.

Finally, to analyze whether blocking ErbB-3 has an effect also on colon cancer growth *in vivo,* we generated xenografts of Co123 and CC09. As shown in Figure 4 treatment with EV20 significantly delayed the outgrowth of these cancers. Importantly, this effect was observed when the antibody was administered immediately after cell engraftment (Figure 4A), but also when tumors were already established (Figure 4B), thus suggesting that anti-ErbB3 therapy may be useful both to limit CSC-induced initiation and to prevent tumor growth of established tumors.

## DISCUSSION

In the past twenty years, development and approval of targeted therapeutics, in particular monoclonal antibodies Bevacizumab, Cetuximab and Panitumumab (anti-VEGF and anti-EGFR, respectively), have significantly prolonged median survival of patients with metastatic CRC [15-21]. However, approximately 50% of metastatic CRC present with *KRAS* mutations and as a consequence anti-EGFR therapies are not effective [22]. In addition, half of the patients with wild-type *KRAS,* which in principle should be responsive to these targeted therapeutics, do not display benefit of the treatment. Mounting evidences suggest that *BRAF* mutations, occurring in 8-10% of CRC patients [23], may be in part responsible for this lack of response [24]. Accordingly, *BRAF* mutations observed in metastatic colorectal cancer patients are associated with a dramatic increase in mortality, compared to those with tumors with wild-type *BRAF* [25]. Mutations in *BRAF* have been documented in several human malignancies, including thyroid, ovarian cancer and melanoma where they appear to play an important role [26]. For this reason in the recent past a plethora of BRAF inhibitors have been developed and tested in preclinical models. Among them, Vemurafenib, a potent and selective small molecule inhibitor of BRAF-V600E (the most frequent mutated form of BRAF), which has been approved by U.S. Food and Drug Administration (FDA) and European Medicines Agency (EMA) for the treatment of metastatic melanoma, showed a significant clinical response [7]. Unfortunately, clinical trials using this drug on patients with metastatic CRC harboring the BRAF-V600E mutation have given disappointing results showing a very low percentage of clinical response (about 5%) [9]. In recent studies, it has been proposed that EGFR-mediated reactivation of the PI3K and MAPK pathways leads to resistance to Vemurafenib in CRC cells [14, 27]. In fact, high expression levels of EGFR are observed in CRC, but not in melanoma cells, explaining the different response of these two tumors. All these data highlight the importance of the tumor microenvironment and in particular of growth factors that may be secreted by tumor-associated fibroblasts.

NRG-1β, the major ligand for ErbB-3, promotes ErbB-2/ErbB-3 dimerization mainly leading to the activation of PI3K/AKT and MEK/ERK signaling pathways [28]. Up-regulation of this signaling axis has been documented in several types of cancer, including breast, lung, pancreas and melanoma [2]. Moreover, in melanoma, thyroid and CRC the use of RAF and ERK inhibitors has been shown to result in removal of the feedback inhibition exerted on ErbB-3 transcription. In other words, inhibition of BRAF-V600E may relieve a transcriptional inhibition and up-regulate receptor expression, potentially allowing escape from Vemurafenib-based therapies [27, 29, 30]. Different clinical and animal studies have shown that NRG-1β is secreted in the tumor microenvironment and plays a crucial role in tumor progression and therapy resistance and that overexpression of this ligand is associated with worse clinical outcome [31, 32].

We show that both ErbB-2 and ErbB-3 receptors are expressed in colon CSCs and that NRG-1β plays an important role in sustaining proliferation of both wild-type and BRAF-V600E mutated cells. This data extends previous reports that pointed to a role of ErbB-3 in CRC progression [12]. Here we clearly provide evidence that the NRG-1β/ErbB-3 axis can induce the cancer stem cell compartment in tumors and induces their clonogenic capacity even in BRAF-V600E mutant CSCs. Interestingly, despite the fact that BRAF-V600E cells are less dependent on growth factors as compared to the wild-type counterpart, their growth can be significantly stimulated by NRG-1β. This stimulation does not appear to be due to an autocrine loop as we did not observe production of the ErbB-3 ligand in these cell lines (data not shown). Nevertheless, we found that exogenous NRG-1β is able to stimulate colon CSCs and can even rescue the anti-proliferative effects of Vemurafenib through the activation of ErbB-3. In vivo, the source of NRG-1β is likely to be the stromal compartment within tumors and in agreement blocking the activation of ErbB-3 significantly impacts the *in vivo* growth of colon cancers. The blockage of growth is however not complete suggesting that other growth factors, such as EGF can substitute for ErbB-3 activation.

Our data highlight a distinct escape mechanism by which BRAF-V600E mutant colon cancers escape from Vemurafenib blockade. Previous data indicated that EGFR activation could support the outgrowth. Our current data indicate that ErbB-3 activation provides a similarly potent rescue mechanism. Our data therefore indicate that a general ErbB-family inhibition is potentially required to circumvent the escape mechanisms activated upon BRAF inhibition.

## MATERIALS AND METHODS

### Colon cancer stem cells culture and reagents

Colon cancer tissues were collected according to the standard medical ethical procedures from Academic Medical Center or University of Palermo. Patient-derived colon cancer stem cells were generated and cultured as previously described [33, 34]. Briefly, colon cancer stem cells were maintained in Dulbecco's modified Eagle's medium/Ham's F12 nutrient mixture (DMEM/F12) supplemented with N2 supplement (Life Technologies, Paisley, UK), 20 ng/ml epidermal growth factor, 10 ng/ml basic fibroblast growth factor (Peprotech, Rocky Hill, NJ) at 37°C in 5% CO2 humidified incubator. All cultures were passaged by enzymatic dissociation using trypsin and trypsin inhibitor (Sigma-Aldrich Corporation, St. Louis, MO, USA). For stable ErbB-3 silencing, cells were infected with pSuper retro–based vectors as described [35]. Control vector pSuper 4Mut contains a four-point mutated sequence unable to target the human ErbB-3 mRNA [36]. NRG-1β was purchased from R&D (R&D Systems, Inc., MN, USA) or Cell Signaling Technology (Danvers, MA, USA). Vemurafenib was purchased from Selleck Chemicals (Houston, TX 77054 USA). EV20 antibody was produced as described [37, 38].

### Microarray data collection and preprocessing

All RNA, treated with 1μM of Vemurafenib or control (DMSO) for 48 hours, were collected using NucleospinRNA kit (Bioke, Leiden, The Netherland). Hybridization was performed to a Gene Chip HT HG-U133+s PM Array Plate (Affymetrix Inc, Santa Clara, California) according to manufacturer's instruction. The gene expression data were normalized and log2 transformed using robust multi-array average (RMA) implemented in the affy package for R [39]. Whenever genes were represented by multiple probesets, the probeset with the highest mean expression was used in subsequent analyses.

### Limiting dilution assays (LD)

The frequency of stem cells was calculated using the maximum-likelihood estimation method of limiting dilution [40]. Cells were plated in serial dilution (1,2,4,8,16,24,32,48 and 64 cells per well) in 96-wells microplate with flat bottom and repellent surface for low attachment (CELLSTAR Cell-Repellent Surface, Greiner Bio-One, UK). After 3 weeks, the number of the clones was counted and statistically evaluated using the Extreme Limiting Dilution Analysis (ELDA) software. Graph showed the means and standard deviation of the observed percentage of the clonogenic population.

### Flow cytometry analysis (FACS)

FACS analysis was performed according to the standard procedures. The following antibodies were used: mouse anti-human EGFR, clone H11 (DAKO), anti-ErbB-2 Affibody^®^ molecule, fluorescein conjugated (Affibody, Solna, Sweden), mouse anti-human ErbB-3, clone SGP1 (Abcam, Cambridge, UK), mouse anti-human ErbB-4, clone H4.77.16 (Abcam), APC goat anti-mouse Ig (BD Bioscience, Oxford, UK).

### Cell proliferation assays

Cells were seeded into 96-wells microplate (2500 cells/well) and cell proliferation over mentioned time period was determined using Cell Titer Blue from Promega (Madison, WI 53711 USA) according to the manufacturer protocol. Briefly, 20 μl of reagent was added into each 100 μl of medium and incubated for 4 hours in 37°C. The fluorescence was then measured in microplate reader (Tecan) with excitation 535 nm and an emission of 590 nm. Fluorescence reading was normalized against the empty well and proliferation was plotted as histograms or curves with data points showing mean ±SD.

### Immunochemistry

Lysates from cells in culture were prepared by washing cells twice in cold PBS followed by lysis with either HNTG buffer (50mM HEPES pH 7.5, 150mM NaCl, 10% glycerol, 1% Triton X-100, 5mM EGTA) or RIPA lysis buffer supplemented with protease and phosphatase inhibitors (Sigma-Aldrich Corporation). Immunoblotting was performed as described [41]. The following antibodies were obtained from Cell Signaling Technology: phosphorylated ErbB-3 (Tyr1289), phosphorylated AKT (Ser473), phosphorylated ERK1/2 (Thr202/Tyr204), AKT and ERK1/2. Anti ErbB-3 was sourced by Santa Cruz Biotechnology (Santa Cruz, CA, USA). Anti-Actin was from Sigma-Aldrich Corporation.

### Animals

Athymic nude-Foxn1^nu^ mice were obtained from Harlan Laboratories (San Pietro Al Natisone, UD 33049, Italy), aged 5-7 weeks and weighting approximately 20-25 g. Animals were maintained under specific pathogen-free conditions with food and water provided *ad libitum* and the animals' health status was monitored daily. Procedures involving animal and their care were established according to the institutional guidelines in compliance with national and international policies.

### qRT-PCR

Total RNA was isolated using NucleospinRNA kit (Bioke, Leiden, The Netherland) according to the supplier's instructions. RT was performed on 1 μg RNA using Supercript III reverse transcriptase (Invitrogen, Carlsbad, CA, USA). After denaturation at 95°C for 2 min, PCR was performed for 40 cycles (1 min at 95°C, 45 s at 63°C, 45 s at 72°C) using SYBR Green (Roche). Transcript levels were compared relative to b-Actin housekeeping genes using the following primers: ACTB-Fwd CAGAAGGATTCCTATGTGGGCGA; ACTB-Rev TTCTCCATGTCGTCC

CAGTTGGT; EGFR-Fwd GTGATCCAAGCTGTCCCAAT; EGFR-Rev ACTGGTTGTGGCAGC

AGTC; ERBB2-Fwd TGTGTGGACCTGGATGACAA; ERBB2-Rev GATGAGGATCCCAAAG

ACCA; ERBB3-Fwd TGGGGAACCTTGAGATTGTG; ERBB3-Rev GAGGTTGGGCAATGGTAGAG

### *In vivo* tumor growth

CC09 (2×10^5^) or Co123 (1×10^6^) cells were resuspended in matrigel (in a ratio 1:6 with calcium and magnesium-free PBS) and injected subcutaneously into the right flank of mice. We used two xenograft models. In the first one treatment with the antibody (EV20 at the dose of 10 mg/kg, twice a week) was initiated 1 week after cells engraftment when tumors were not yet established. In an other set of experiments we used a second xenograft model in which mice received the antibody treatment once tumors had reached the approximate size of 100 mm^3^. Animals were divided in two arms in a manner to provide a similar range of tumor size in each group. The test groups received intraperitoneal injections of EV20 (10 mg/kg) twice a week, whereas the control groups received PBS only. Tumor volumes were monitored twice a week by a caliper and volumes were calculated using the following formula: tumor volume = (length * width^2^)/2. Results are expressed as Relative Tumor Volume, i.e. [(measured volume time point x) / (volume at start of treatment)]. *p* values were determined by Student's *t* test and considered significant for *p* < 0.05.

### Statistical analysis

Statistical analyses were done using R version 3.1.2 [42] and Bioconductor 3.0 [43]. The global effects of Vemurafenib treatment on gene expression were analyzed using the limma package [44], modeling the cell line and treatment effects. *P* values were adjusted for multiple testing using Benjamini-Hochberg FDR correction. Genes were considered to be differentially expressed when *p* < 0.05. The resulting list of differentially expressed genes was analyzed for functional enrichment using Gene Ontology [45] and the Panther Classification System [46]. *P* values were adjusted for multiple testing with the Bonferroni correction.

## SUPPLEEMENTARY MATERIAL FIGURES


