# Supplementary material for: ErbB-3 activation by NRG-1β sustains growth and promotes vemurafenib resistance in BRAF-V600E colon cancer stem cells (CSCs)
Source: Oncotarget. 2015 Jun 25;6(19):16902–11. doi: 10.18632/oncotarget.4642 (PMC4627280; doi:10.18632/oncotarget.4642)
Supplement: Supplementary file 1 [file oncotarget-06-16902-s001.pdf]

ErbB-3 activation by NRG-1β sustains growth and promotes vemurafenib resistance in BRAF-V600E colon cancer stem cells (CSCs)

Supplementary Material

A

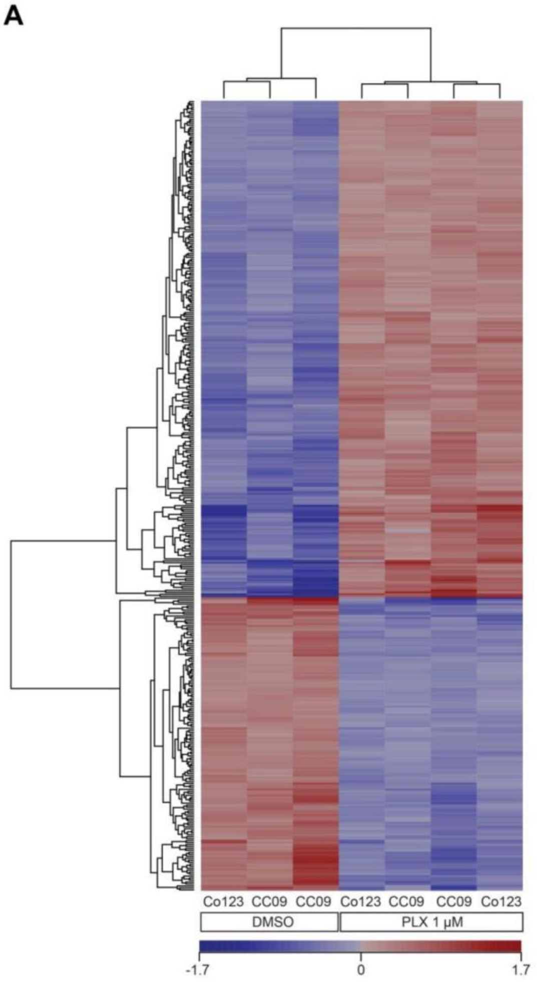

B

CATEGORY	Adjusted P
**Down regulated genes**	
condensed chromosome, centromeric region (GO:0000779)	6,00E-09
condensed chromosome kinetochore (GO:0000777)	5,45E-08
chromosome, centromeric region (GO:0000775)	1,71E-07
kinetochore (GO:0000776)	6,09E-07
chromosomal region (GO:0098687)	6,52E-07
organelle organization (GO:0006996)	7,14E-07
mitotic nuclear division (GO:0007067)	7,79E-07
organelle fission (GO:0048285)	2,68E-06
cell cycle (GO:0007049)	3,02E-06
mitotic cell cycle (GO:0000278)	3,67E-06
condensed chromosome (GO:0000793)	7,08E-06
nuclear division (GO:0000280)	7,29E-06
mitotic cell cycle process (GO:1903047)	1,80E-05
cell cycle process (GO:0022402)	2,38E-05
single-organism organelle organization (GO:1902589)	4,13E-05
chromosomal part (GO:0044427)	1,23E-04
cell division (GO:0051301)	1,51E-04
mitotic prometaphase (GO:0000236)	2,27E-04
spindle (GO:0005819)	5,02E-04
chromosome (GO:0005694)	9,11E-04
spindle pole (GO:0000922)	1,57E-03
regulation of chromosome segregation (GO:0051983)	3,23E-03
mitochondrion (GO:0005739)	3,64E-03
mitotic cell cycle phase (GO:0098763)	6,05E-03
cell cycle phase (GO:0022403)	6,05E-03
biological phase (GO:0044848)	6,96E-03
chromosome organization (GO:0051276)	8,63E-03
microtubule organizing center (GO:0005815)	1,03E-02
centrosome (GO:0005813)	1,32E-02
CENP-A containing nucleosome assembly (GO:0034080)	1,38E-02
CENP-A containing chromatin organization (GO:0061641)	1,38E-02
chromatin remodeling at centromere (GO:0031055)	1,90E-02
M phase (GO:0000279)	2,17E-02
mitotic M phase (GO:0000087)	2,17E-02
mitochondrial part (GO:0044429)	2,99E-02
centromere complex assembly (GO:0034508)	3,41E-02
**Up regulated genes**	
membrane-bounded vesicle (GO:0031988)	7,72E-03
vesicle (GO:0031982)	9,91E-03
endomembrane system (GO:0012505)	2,04E-02
extracellular vesicle (GO:1903561)	4,58E-02
extracellular exosome (GO:0070062)	4,58E-02
extracellular organelle (GO:0043230)	4,63E-02
extracellular membrane-bounded organelle (GO:0065010)	4,63E-02
extracellular region part (GO:0044421)	4,82E-02

C

Gene	logFC	Average Expression	Adjusted P
ERBB3	0,43	11,12	0,046
EGFR	0,30	7,48	0,126
ERBB2	0,25	8,71	0,146
ERBB4	-0,04	3,13	0,801

D

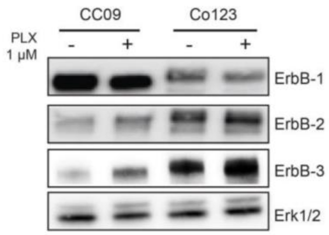

Supplementary Fig. S1.

(A) Heatmap depicting differential gene expression in DMSO and 1 μM Vemurafenib treated BRAF mutant cells using Affymetrix U133 array ($p < 0.05$). Up-regulated genes depicted in red, down-regulated genes depicted in blue (see color bar). **(B)** Gene Ontology (GO) analysis of the differentially expressed genes in A revealed a number of gene clusters that are associated with the response to Vemurafenib. **(C)** Microarray data analysis on ErbB-family receptor gene expression showed the significant upregulation of ErbB-3 after Vemurafinib exposure. LogFC indicates the change in expression upon Vemurafinib treatment compared to DMSO (log2 ratio), average expression is the mean log2 intensity from all measurements **(D)**Immunoblot analysis of total ErbB-1, ErbB-2 and ErbB-3 in BRAF mutant cells treated with 1μM Vemurafenib for 48 hours. Total Erk1/2 was used as loading control.

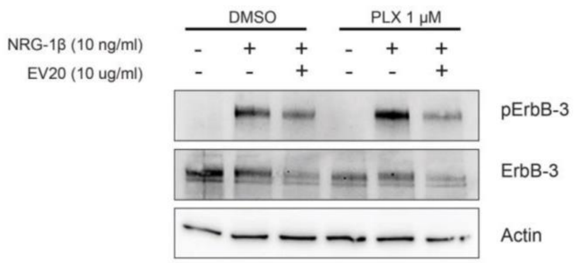

Supplementary Figure S2.

Immunoblot analysis of ErbB-3 phosphorylation of Co123 cells treated overnight with Vemurafenib (1µM) and then stimulated for 5 minutes with NRG-1β (10ng/ml) in presence or absence of EV20 (10 µg/ml).
